# Volar locking plate fixation for distal radius fractures: did variable-angle plates make difference?

**DOI:** 10.1007/s00264-022-05469-z

**Published:** 2022-06-11

**Authors:** Mohamed Abdel-Wahed, Ahmed Abdel-Zaher Khater, Mahmoud Ahmed El-Desouky

**Affiliations:** 1 Department of Trauma and Orthopedics, Faculty of Medicine, Cairo University, Cairo, Egypt; 2 Ahmed Maher Teaching Hospital, Cairo, Egypt

**Keywords:** Distal radius fractures, Volar locking plate, Polyaxial, Fixed angle, Variable angle

## Abstract

**Purpose:**

Two different locking plate designs are now being used for volar plating of the distal radius fractures based on the freedom of screw direction; the fixed-angle, and the variable-angle (polyaxial) plates. We investigated the clinical and radiographic outcomes of both designs.

**Methods:**

We reviewed 96 patients with 113 unstable distal radius fractures that were operated on with volar locking plates. The patients’ mean age was 41 years. Fixed-angle volar locking plates were utilized in 65 fractures and variable-angle volar locking plates in 48 fractures through modified Henry approach or extended carpal tunnel approach. Full clinical and radiographic evaluation was done for all patients with a mean follow-up of 14 months.

**Results:**

All patients had acceptable clinical and radiographic parameters. The overall functional results (Mayo score, Quick Disability of Arm, Shoulder, and Hand (Q-DASH) score, Range of motion (ROM), and grip strength) were in favor of the variable-angle plate. The radiographic parameters were better with the variable-angle group. The variable-angle group recorded less operative time but more mean image intensifier exposure time. There were two cases of flexor tendon rupture with the fixed-angle group. Fixation with the fixed-angle system needed K-wire augmentation more than the variable-angle group. There was a positive correlation between hand dominance and the final score.

**Conclusion:**

Distal radius volar locking plates yield satisfactory results comparable among different designs. In our series, the variable-angle system showed slightly better function and radiographic outcomes. Supplementary K-wires were needed more frequently with the fixed-angle system.

## Introduction

Distal radius fractures represent about one-sixth of all fractures seen in the emergency department [1]. While many of these fractures can be successfully managed conservatively, others require surgical stabilization. Treatment choice is debatable in unstable intra and extra-articular fractures. The most popular stabilization techniques include external fixation, pinning, dorsal or volar plating, or a combination of these methods [2].

Volar plating solved a lot of problems and is now the most commonly used method for surgical stabilization of distal radius fractures [3]. Volar locking plates mechanically bridge the fracture acting as a load-bearing implant (internal fixator) with a lower incidence of failure. The subchondral placement of the distal screws is essential to support the articular surface and prevent loss of reduction. Experimental biomechanical evidence supports the use of volar plating with dorsally comminuted unstable distal radius fractures [4, 5].

Classically, the volar-locking plates were designed with fixed-angle locking screws. The fixed direction of the locked screws rendered this plate unforgivable to minor errors of implant application, with a risk of screw penetration. Moreover, some fragments (especially dorsal) could not be easily captured by the screws, with the subsequent need to apply added hardware to ensure stabilization of all fracture fragments [6, 7].

**Fig. 1:**
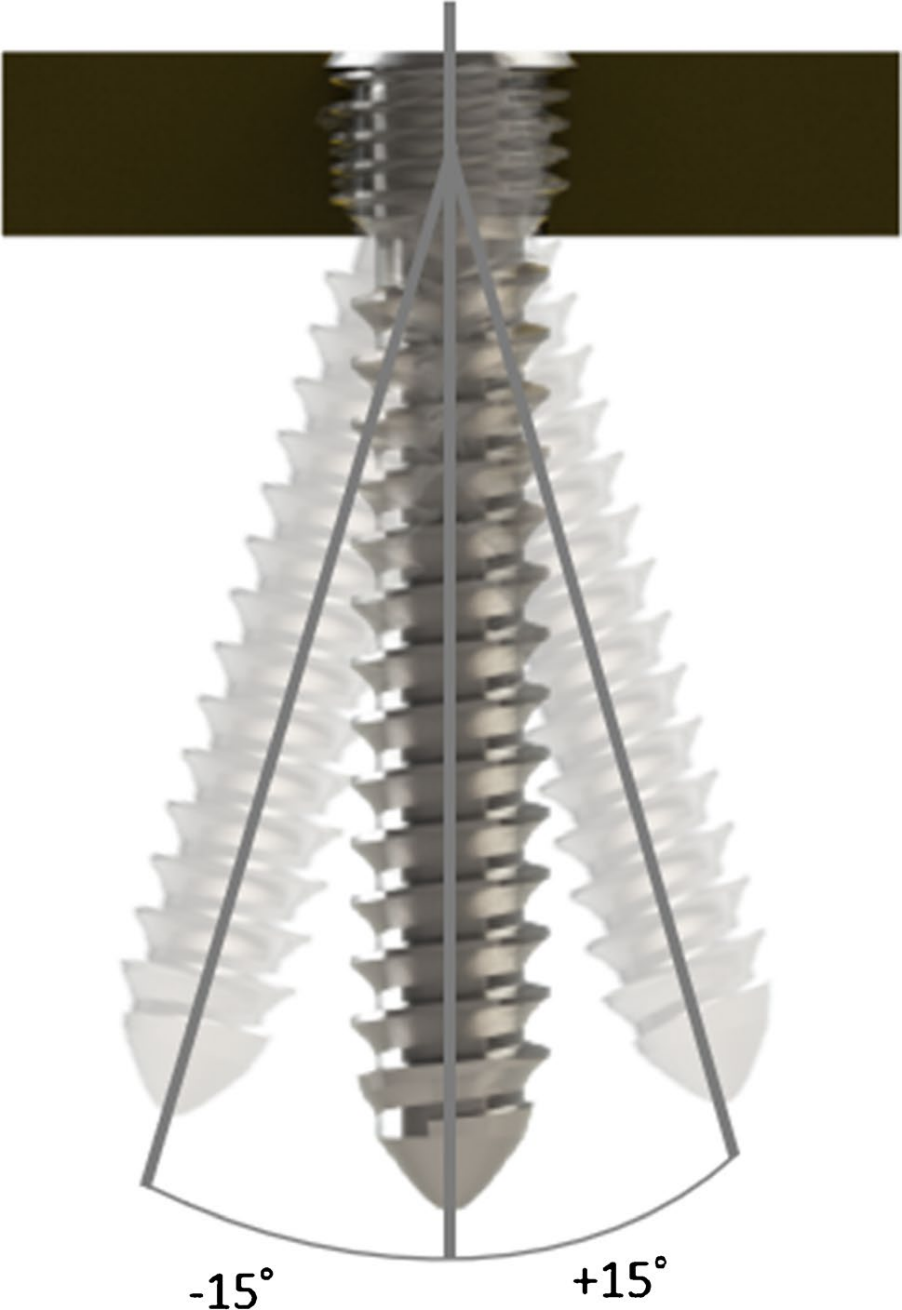
Locking screw direction variation in variable-angle locking plate

More recently, the volar plates are produced with a variable-angle locking mechanism. The presumed advantage is the flexible positioning of both the plates and the screws to accommodate variations in fracture lines while still minimizing the risk of screw perforation of either the distal radio-ulnar joint or the radio-carpal joint. In addition, the free screw direction can be adapted to specific fracture fragments (Fig. 1). Furthermore, there is a large variability in the arc of screw coverage that can be achieved. In addition to all these advantages, they still maintain angular stability [7–9].

In this study, we compared the functional and radiological outcomes of comminuted distal radial fractures operated with the fixed-angle versus variable-angle volar locking plates.

### Patients and methods

This is a retrospective study conducted at a tertiary care academic institute. The records of patients operated for comminuted fractures at the distal end of radius presented to the causality department over three years were revised (2017, 2018, 2019). *The scientific and ethical board reviewed and approved the protocol before study initiation. It was performed following the ethical standards of the 1964 Declaration of Helsinki.*

**Fig. 2:**
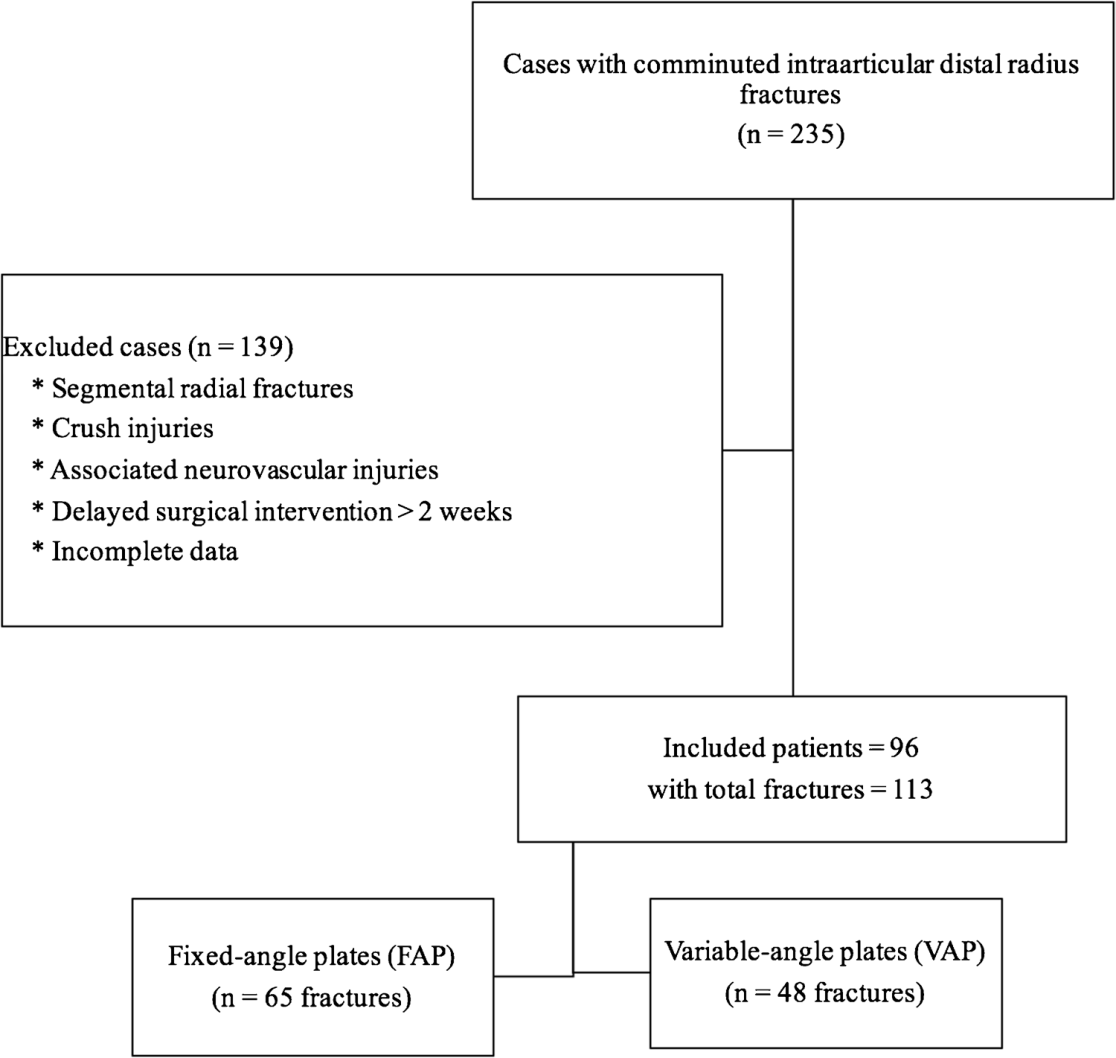
Flow chart of included cases

We included skeletally mature patients with comminuted intra-articular distal radial fractures AO type 23-C, who completed at least nine months of follow-up period. During the study period, 235 patients were identified. We excluded cases with segmental fractures of radius, associated carpal injuries, high-grade open fractures (crush injuries), cases with associated neurovascular injuries, cases with a delay of surgical intervention more than two weeks, and cases with incomplete data. Available for analysis after exclusions were 96 patients with 113 fractured distal end radius; 65 fractures were managed with fixed-angle plates (FAP), and the other 48 were managed with variable-angle plates (VAP) (Fig. 2).

The mean age of patients included in this study was 41.28 years (range from 22 to 60 years). There was a significant difference in the age of cases in both groups as the mean age in the FAP group was 44.7 years while in the VAP group it was 36.3 years (*p* < 0.001). There were 72 males (75%) and 24 females (25%) with 70 patients fractured their dominant side (72.9%), nine fractured their non-dominant side (9.4%), and 17 patients fractured both sides (17.7%). Fall on outstretched hand (FOOSH) was the commonest mode of trauma in 45 patients (46.9%), while 18 patients sustained fracture after direct trauma (18.8%), 21 patients after road traffic accidents (RTA) and motor vehicle accidents (MVA) (21.9%), and 12 patients after falling from height (FFH) (12.5%). Interval lag time before fracture fixation ranged from a few hours to 15 days. Apart from mean age, there was no statistically significant difference between the demographic features of both groups (Table 1).

**Table 1:** Demographic features and methodology of included patients

		Fixed-angle plate (FAP)		Variable-angle plate (VAP)		P value	Total	
		Count	%	Count	%		Count	%
Sex	Female	13	22.8%	11	28.2%	0.549	24	25%
	Male	44	77.2%	28	71.8%		72	75%
Mode of trauma	Direct trauma	9	15.8%	9	23.1%	0.315	18	18.7%
	FFH	7	12.3%	5	12.8%		12	12.5%
	FOOSH	31	54.4%	14	35.9%		45	46.9%
	MVA and RTA	10	17.5%	11	28.2%		21	21.9%
Affected side	Dominant	45	78.9%	25	64.1%	0.312	70	72.9%
	Non-dominant	4	7.0%	5	12.8%		9	9.4%
	Bilateral	8	14.0%	9	23.1%		17	17.7%
Approach	Modified Henry	49	75.4%	45	93.8%	0.010	94	83.2%
	Extended carpal tunnel	16	24.6%	3	6.2%		19	16.8%
Pronator quadratus	Stripping	50	76.9%	34	70.8%	0.464	84	74.3%
	Preservation	15	23.1%	14	29.2%		29	25.7%
Supplementary K-wires	Used	18	27.7%	3	6.3%	0.004	21	18.6%
	Not used	47	72.3%	45	93.7%		92	81.4%
Ulnar styloid fixation	Needed	9	13.8%	6	12.5%	0.835	15	13.3%
	Not needed	56	86.2%	42	87.5%		98	86.7%

^**^ The first 3 items are calculated for the total number of patients = 96

The others are calculated for the total number of fractures = 113

All patients admitted to the causality department follow the trauma protocol of the hospital with precise history taking, clinical, and radiological assessment including CT scans on the affected wrist and a preliminary reduction in a below elbow slab. Patients were scheduled for surgery on the nearest operative list unless there is acute median nerve entrapment or open injury.

The approach and plate choice depended on the surgeon’s judgment and implants available at the time of surgery. The modified Henry approach [10] was utilized in 94 fractures’ fixation, while the extended carpal tunnel approach [10] was utilized in the other 19. Subperiosteal dissection of the pronator quadratus muscle through an L-shaped incision was done to expose the fracture site in 84 fractures while the pronator quadratus preservation was adopted in 29 fractures.

### Implants used

**Fig. 3:**
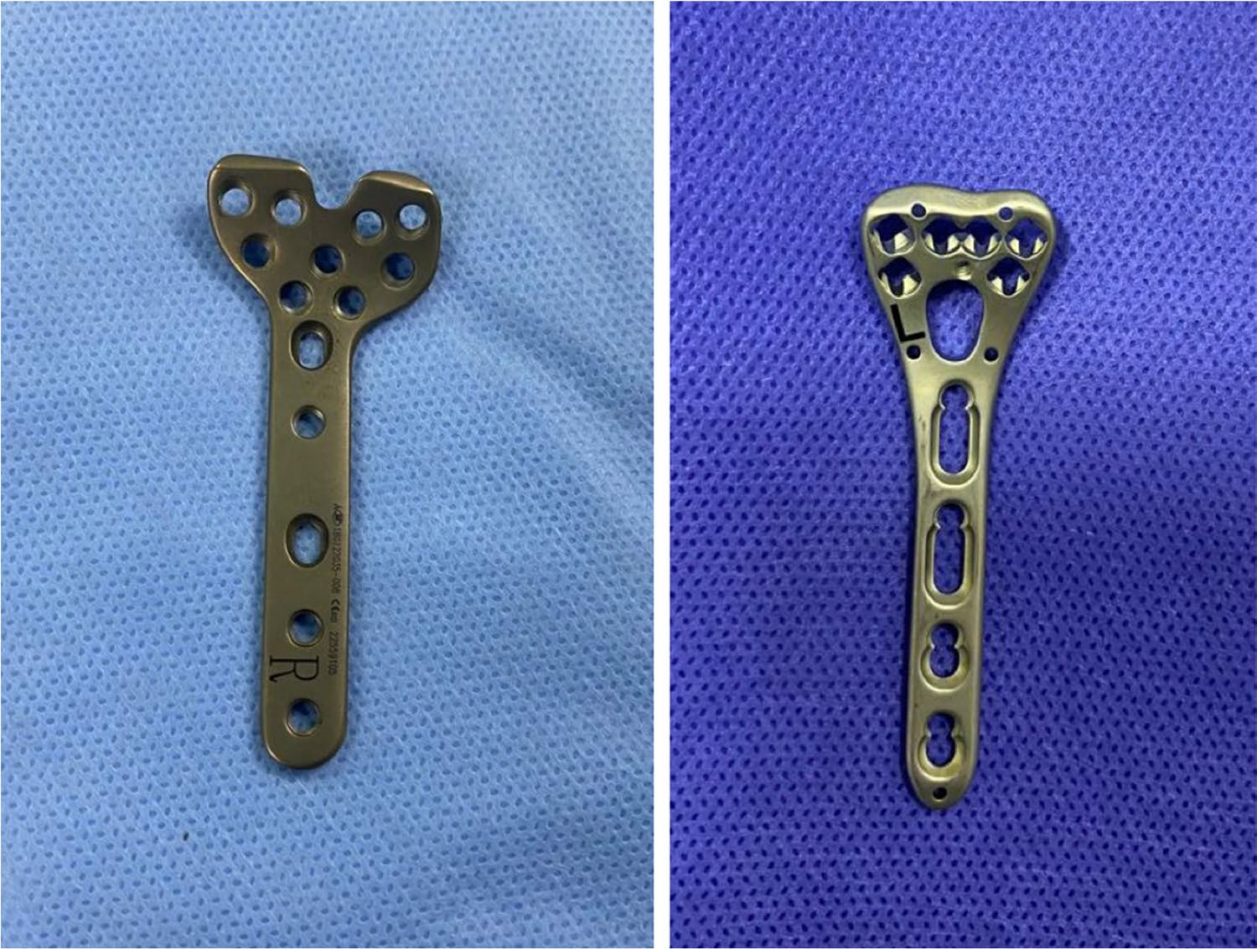
Fixed-angle and variable-angle locking plates used in the included cases

The VAPs used in the study were Zimmer Biomet 2.7 mm and Medtronic 2.4 mm. The FAPs were Medtronic Distal Radius Volar Column plates I, II 2.7 mm (Fig. 3).

K-wire augmentation to fix styloid fragment or dorsal fragment was needed whenever the plate and screws weren’t catching all the fragments. This was needed in 18 cases managed with FAP (27.7%), and in only three cases fixed with the VAP system (6.3%).

The situation of associated ulnar styloid fractures necessitating fixation was encountered in nine patients in which the FAP was utilized, and in six patients in the VAP group. In these 15 patients, headless compression screws were used in seven cases, hooked plate in two cases, and tension band wiring in the other six cases.

Post-operatively, all patients were immobilized in a dorsal slab for two weeks except patients with augmentation K-wires, who were immobilized for six weeks. After removal of the slab, patients were allowed to do non-loaded activities of daily living including eating and personal care with a part-time wrist brace. A rehabilitation program was continued under physiotherapists’ supervision.

Radiographs were done on the sixth week to evaluate fixation and signs of radiological union and repeated at the 12th week. Additional imaging was ordered according to the clinical improvement upon need.

**Fig. 4:**
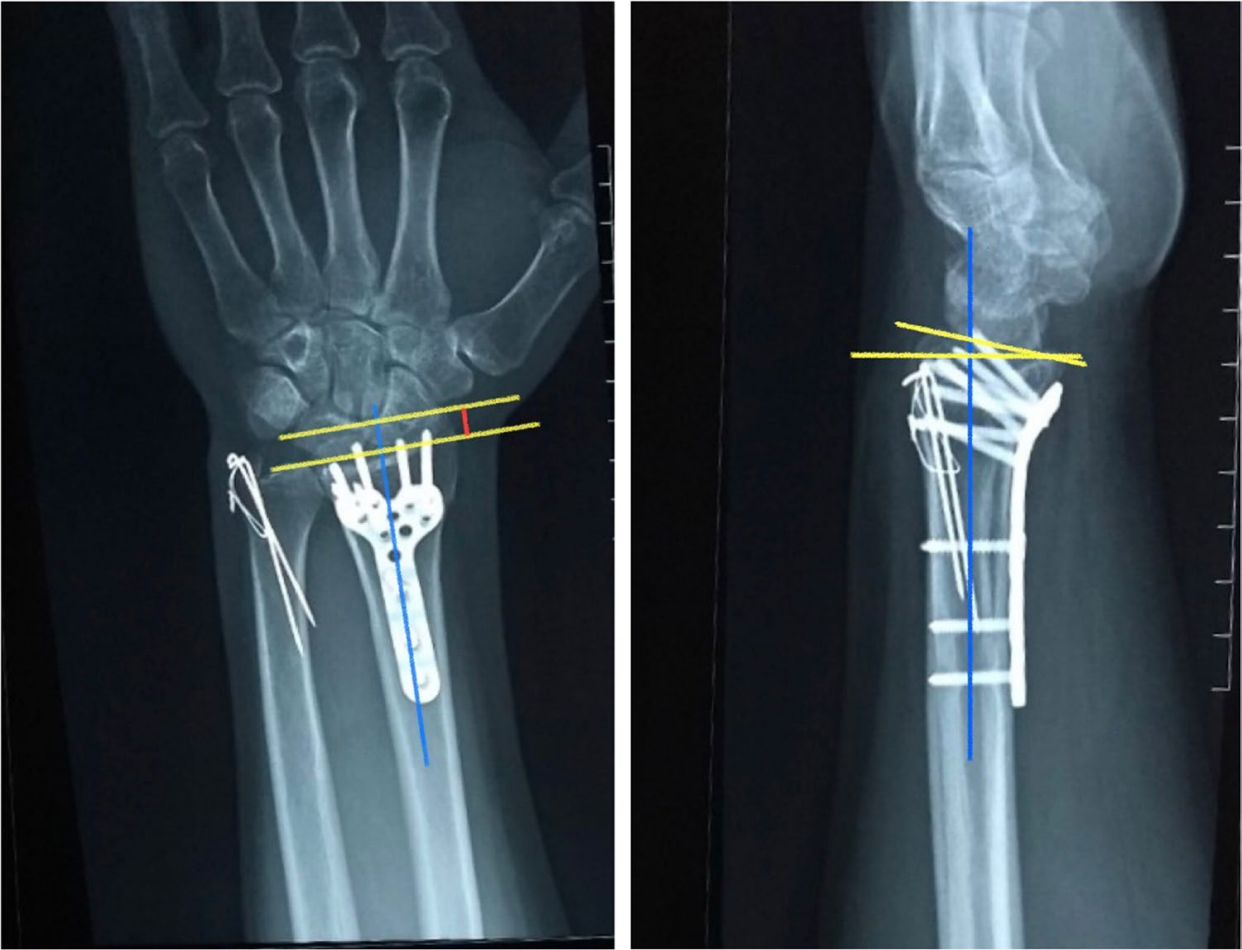
Radial height and volar tilt measured in a case fixed with a fixed-angle volar locking plate

**Fig. 5:**
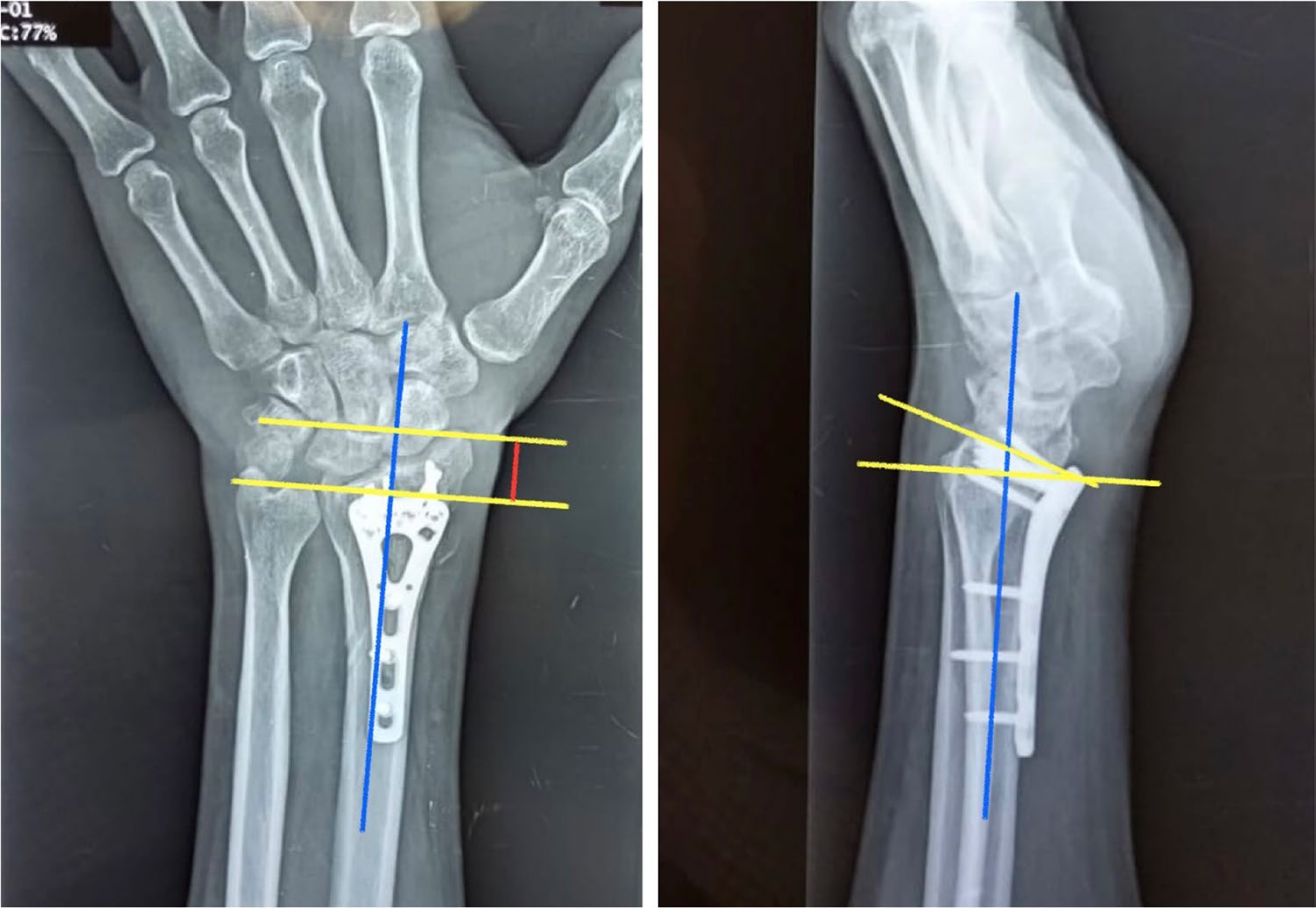
Radial height and volar tilt measured in a case fixed with a variable-angle volar locking plate

Clinical and radiographic re-evaluation was done every three months for uneventful cases including subjective clinical outcomes (Mayo, and Q-DASH scores, the validated Arabic form was used), objective clinical outcomes (wrist ROM and grip strength), and measuring radiographic parameters (radial height and volar tilt) (Figs. 4 and 5). Cases that developed any complaints during the follow-up period had a more frequent assessment.

Assessment of all candidates was done by a single assessor. The ROM was measured using a goniometer (**Baseline** plastic goniometer, 30 cm), and the grip strength was measured using a Jamar hydraulic hand dynamometer (model 5030J1).

### Statistical methods

Data were summarized using mean and standard deviation for quantitative variables or count and percentages for categorical variables. Comparisons were done using unpaired *t* test or Chi-square (*χ*^2^) tests. *P* values less than 0.05 were considered statistically significant. SPSS 28 was used.

## Results

The included patients had a minimum follow-up period of nine months, with a mean follow-up period of 14 months (range 9–28). The operative time for all cases ranged between 37 and 105 minutes. The mean operative time in the FAP group was 80 min, while it was 72 in the VAP group. The fluoroscopy exposure time for all cases ranged from 27 to 315 seconds. Its mean value in the FAP group was 82 s, while in the VAP group it was 113 s.

Cases showed radiographic union starting at the third-month follow-up radiographs. Twenty-three cases had to wait for the sixth-month follow-up radiographs. No case had a nonunion of their fracture.

Analysis of the ROM demonstrated superior results in the VAP group with statistical significance for wrist extension, ulnar, and radial deviation, but insignificant for wrist flexion. Grip strength measured 20.58 kg for the VAP group compared to 16.28 kg in the FAP group (*p* < 0.001). MAYO and Q-DASH scored superior results in the VAP group (*p* < 0.001 in both scores). The radial height and volar tilt were better in the VAP group compared to the FAP (*p* < 0.001 for both parameters) (Table 2).

**Table 2:** Clinical and radiographic results of cases

		Fixed-angle plate (FAP)		Variable-angle plate (VAP)		*P* value	Total	
		Mean	SD	Mean	SD		Mean	SD
Range of motion	Wrist flexion	42.55	11.42	45.96	9.43	0.095	44.00	10.71
	Wrist extension	39.85	7.24	55.60	10.62	< 0.001	46.54	11.77
	Radial deviation	12.38	1.94	15.29	2.41	< 0.001	13.62	2.59
	Ulnar deviation	25.46	5.47	35.90	4.00	< 0.001	29.89	7.12
Functional outcome	Grip strength (Kg)	16.28	4.03	20.58	4.50	< 0.001	18.11	4.73
	VAS	2.25	1.71	1.73	1.30	0.082	2.03	1.56
	Mayo	70.45	11.85	80.31	5.99	< 0.001	74.64	10.92
	Q DASH	9.46	6.63	2.76	2.46	< 0.001	6.61	6.23
Radiographic parameters	Radial height	6.34	2.71	8.75	1.77	< 0.001	7.36	2.64
	Volar tilt	6.92	3.05	10.02	1.67	< 0.001	8.24	2.97

We compared the results of patients younger than 50 years in both groups and found better outcome in the VAP group in all parameters. Apart from wrist flexion and VAS score, all differences were statistically significant (Table 3). When comparing the results of cases younger than 50 years with those equal or more than 50, all parameters showed better improvement in younger age group which was significant in radial and ulnar deviation, Mayo score, Q-DASH score, radial height, and volar tilt (Table 3).

**Table 3:** Comparison of clinical and radiographic outcome in special groups

		Cases without associated Ulnar styloid fracture (*n* = 92)			Cases younger than 50 years (*n* = 70 cases or 75 fractures)			Comparison between age groups		
		Fixed-angle plate (FAP) (*n* = 52)	Variable-angle plate (VAP) (*n* = 40)	*P* value	Fixed-angle plate (FAP) (*n* = 43)	Variable-angle plate (VAP) (*n* = 27)	*P* value	< 50 years (*n* = 70)	≥ 50 years (*n* = 26)	*P* value
Range of motion	Wrist flexion	42.73	45.52	0.167	43.22	46.14	0.208	44.61	41.84	0.255
	Wrist extension	39.55	54.19	< 0.001	40.28	55.43	< 0.001	47.51	43.12	0.100
	Radial deviation	12.37	15.07	< 0.001	12.70	15.21	< 0.001	13.90	12.64	0.031
	Ulnar deviation	25.64	36.26	< 0.001	25.91	35.83	< 0.001	30.65	27.24	0.034
Functional outcome	Grip strength (Kg)	16.13	20.64	< 0.001	16.26	20.62	< 0.001	18.34	17.28	0.324
	VAS	2.20	1.69	0.100	2.04	1.74	0.336	1.90	2.48	0.100
	Mayo	70.07	80.00	< 0.001	72.87	80.33	< 0.001	76.43	68.32	0.001
	Q DASH	9.50	2.67	< 0.001	8.37	2.68	< 0.001	5.66	9.98	0.020
Radiographic parameters	Radial height	6.25	8.76	< 0.001	6.78	8.90	< 0.001	7.80	5.84	0.001
	Volar tilt	6.89	9.98	< 0.001	7.46	9.93	< 0.001	8.64	6.84	0.025

Comparing both groups with exclusion of the of cases with associated ulnar styloid fractures (21 cases total; 15 underwent fixation and six cases managed conservatively), better outcome was observed in the VAP group. The results were statistically significant for wrist extension, radial and ulnar deviation, Mayo score, Q-DASH score, radial height, and volar tilt (Table 3).

Generally, there was no significant difference in the complication rate between both groups. In the FAP, four patients suffered manifestations of carpal tunnel syndrome and underwent surgical release in the follow-up period. Two patients suffered delayed wound healing which resolved completely after one month. Three had superficial wound infection and resolved with regular dressings and antibiotic administration for six weeks. One patient had screw misplacement in the radio-carpal joint and other three had dorsally penetrating screws, one of them remained asymptomatic and the other two developed wrist swelling, and screw exchange was selected. Six patients developed complex regional pain syndrome (CRPS), four of them improved partially with an intensive protocol of physiotherapy, and two of them showed full recovery. There were also two cases of flexor tendon rupture who needed a later tendon transfer surgery.

In the VAP group, three patients had manifestations of carpal tunnel syndrome. One patient developed ulnar nerve manifestations in the form of tingling and numbness along the fourth and fifth fingers. One patient showed delayed wound healing with radio-ulnar screw perforation (same patient) which was asymptomatic and wound healing was completed by one month. Two patients had superficial wound infection resolved with regular dressings and antibiotic administration for two weeks. Two patients had dorsally penetrating screws and one of them remained asymptomatic and the other developed wrist swelling and underwent early implant removal. Two patients developed CRPS and improved on an intensive protocol of physiotherapy (Table 3).

Secondary surgery was needed for 25 cases. Seven cases underwent removal of their variable-angle plates (14.6%), while nine cases removed their fixed-angle plates (13.8%). Four cases from the FAP group needed secondary procedure for removal of a buried K-wire that could not be removed in the clinic easily. Three cases underwent secondary surgery to exchange mal-placed screws. Two cases underwent tendon transfer surgery for ruptured flexor tendons.

## Discussion

Emerging industrial implants have always been propulsive for advanced trauma and fracture management. A lot of newly introduced implants minimally survived with practice. Volar locking distal radius plates have stood the test of time as a superior tool for comminuted distal radius fractures with minimal short- and long-term complications. The variable-angle volar locking distal radius plate is a recently used variant of this volar plate. It has the advantage of selective directing the distal screws. The overall judgement on this newer modality is still to be evaluated.

The variable-angle locking plate could be placed proximal to the watershed line while still capable of engaging comminuted distal fragments with deeper insertion of distal screws into the subchondral bone, providing better buttress for the fracture fragments while saving the flexor tendons from late rupture [9].

This work aimed to compare the functional and radiographic outcomes of distal radial fractures operated with the variable-angle versus fixed-angle volar locking plates. A total number of 96 patients suffering 113 fractured distal end radius were retrospectively reviewed and followed up over a mean period of 14 months (range 9–28); 65 fractures managed with fixed angle plates and the other 48 managed with variable angle plates. All patients had acceptable clinical and radiographic parameters. The overall functional results were in favour of the variable-angle plate with slightly better subjective outcomes (Mayo score, and Q-DASH score) and objective outcomes (ROM & grip strength). The variable-angle group recorded less operative time but higher mean fluoroscopic exposure time. Fixation with a fixed-angle system needed K-wire augmentation more than the variable-angle group. The radiological parameters were better with the variable-angle group. Generally, there was no significant difference in the complication rate between both groups.

The variable-angle locking plates have been a matter of research interest lately. Rausch et al. conducted a cadaveric study that supported their biomechanical effectiveness for the management of intra-articular fractures of the distal radius. They found that these plates have higher construct stiffness and superior properties under cyclic loading than fixed-angle plates [11].

Hoffmeier et al., Khatri et al., and Al-Mouazzen et al. reported satisfactory outcomes for the VAP [9, 12, 13]. In their retrospective study, Mehrzad and Kim concluded that the VAP can reduce the rate of hardware-related complications compared to the standard FAP designs while still allowing more flexibility with plate position as well as more uniform fixation of the subchondral surface (Table 4) [14].

**Table 4:** Comparison with the results of other studies

	Khatri et al. [9]	Al-Mouazzen et al. [13]	*Mehrzad and Kim* [14]	Marlow et al. [15]	Seung Cha et al. [16]	Nishiwaki M. et al. [17]	Our study
Study type	Retrospective case series	Retrospective comparative	Retrospective comparative	Retrospective comparative	Retrospective comparative	Prospective comparative	Retrospective comparative
No. of included cases	23 (VAP)	78 FAP = 42 VAP = 36	189 FAP = 60 VAP = 129	107 FAP = 42 VAP = 65	41 FAP = 20 VAP = 21	109 FAP = 54 VAP = 55	113 FAP = 65 VAP = 48
Included fracture types	AO 23-A3 = 4 AO 23-C2 = 9 AO 23-C3 = 10	AO 23- A, B & C	AO 23- A, B & C	AO 23-A = 27 AO 23-B = 8 AO 23-C = 72	AO 23-A = 12 AO 23-B = 2 AO 23-C = 27	AO 23-B3 = 1 AO 23-C1 = 5 AO 23-C2 = 36 AO 23-C3 = 67	AO 23-C
Mean age	32.82 years (19–62)	50.5 years (16–79)	14–92 years	56.1 years in FAP group (18–87) 57.7 years in VAP group (17–92)	61.5 years in FAP group 57.6 years in VAP group	58 years in FAP group 59 years in VAP group	41.28 years (22–60) 44.7 years in FAP group 36.3 years in VAP group
Follow-up period	11.04 ± 2.47 months (6–15)	Minimum 12 months	32–65 months	32.5 months (FAP) 17.2 months (VAP)	8.46 months (FAP) 8.12 months (VAP)	Minimum 12 months	14 months (9–28)
ROM	Flexion = 71.91° Extension = 76.95^o^ Supination = 81.86° Pronation = 94.52^o^			(% Compared to opposite side) Flexion = 82.4% vs 75.6% Extension = 85.8% vs 83.6% Supination = 94.8% vs 86.7% Pronation = 97.9% vs 95.5% RD = 88.3% vs 82.6% UD = 95.7% vs 76.7% *N.B. FAP vs VAP*	Flexion – extension = 51.25 vs 63.50 ^o^ Supination – pronation = 125.5 vs 135.5 ^o^ *N.B. FAP vs VAP*	(% Compared to opposite side) Flexion = 88% vs 85% Extension = 94% vs 94% Supination = 98% vs 98% Pronation = 99% vs 98% RD = 90% vs 94% UD = 92% vs 90% *N.B. FAP vs VAP*	Flexion = 42.55 vs 45.96^o^ Extension = 39.85 vs 55.60^o^ RD = 12.38 vs 15.29^o^ UD = 25.46 vs 35.90^o^ *N.B. FAP vs VAP*
Grip power	94.52% of the opposite side			58.4 kg (FAP) 53.3 kg (VAP)		(% Compared to opposite side) FAP = 91% VAP = 88%	FAP = 16.28 kg VAP = 20.58 kg
Functional scores	Gartland and Werley’s demerit scoring system Excellent (65.22%) Good (34.78%) None of the patients had fair or poor results			VAS FAP = 8.24 VAP = 8.43 Mayo score FAP = 79.44 VAP = 71.83 (VAVLP) Q-DASH score FAP = 21.39 VAP = 20.14	Pain score FAP = 0.57 VAP = 0.63 DASH score FAP = 7.30 VAP = 6.65	PRWE score FAP = 5 VAP = 6 DASH score FAP = 4 VAP = 6	VAS: FAP = 2.25 VAP = 1.73 MAYO score FAP = 70.45 VAP = 80.31 Q-DASH score FAP = 9.46 VAP = 2.76
Radiological parameters	Radial height = 11.84 ± 2.04 mm Radial inclination = 22.89 ± 2.64^o^ Volar tilt = 5.21 ± 5.72^o^ Ulnar variance =—0.29 ± 0.58 mm	Mean percentage of the unsupported subchondral bone significantly lower in the VAP than FAP group (12% vs. 23%, p < 0.001)		Radial height = 8.9 vs 7.3 mm Radial inclination = 22.9 vs 19.5^o^ Volar tilt = 9.1 vs 6.1^o^ *N.B. FAP vs VAP*	Radial height = 11.8 vs 12.3 mm Radial inclination = 22.9 vs 23.2^o^ Volar tilt = 5.6 vs 6.6^o^ *N.B. FAP vs VAP*	Radial height = 11.5 vs 10.8 mm Radial inclination = 23 vs 22^o^ Volar tilt = 8 vs 5^o^ UV = 0.8 vs 0.8 mm *N.B. FAP vs VAP*	Radial length = 6.34 vs 8.75 Volar tilt = 6.92 vs 10.02^o^ *N.B. FAVLP vs VAVLP*
Complications	5 cases (21.7%) Hypertrophic scar (1) Superficial infection (2) Screw misplacement (1) Carpal tunnel syndrome (1)		FAP group: 7/60 (12% had hardware-related complications) VAP group: 0/129 (0% had hardware-related complications)	FAP group: 5/42 = 11.9% Nonunion (1) Malunion (1) Restricted ROM (2) Extensor tendonitis (1) VAP group: 5/65 = 7.7% Carpal tunnel syndrome (1) CRPS (2) Restricted ROM (2)	FAP group: 3/20 = 15% Carpal tunnel syndrome (1) Symptomatic hardware (2) VAP group: 4/21 = 19% Reduction loss (1) Symptomatic hardware (3)	FAP group: 10/54 = 19% Carpal tunnel syndrome (4) Wrist pain (2) Trigger finger (2) DRUJ arthritis (1) Pin site infection (1) VAP group: 21/55 = 38% Wrist pain (4) Carpal tunnel syndrome (3) Trigger finger (3) Intra-articular screws (4) Screw loosening (2) Volar prominence of the plate (2) Deep infection (1) Basilar thumb arthritis (1) Dermatitis (1)	FAP group: 19/65 = 29% Carpal tunnel syndrome (4) Delayed wound healing (2) Superficial infection (3) Screw misplacement (1) Dorsally-prominent screws (3) CRPS (6) Ruptured flexor tendon (2) VAP group: 12/48 = 25% Carpal tunnel syndrome (3) Ulnar tunnel syndrome (1) Delayed wound healing (1) Superficial infection (2) Screw misplacement (1) Dorsally-prominent screws (2) CRPS (2)

A few studies have compared both modalities of distal radius volar locking plates. Unlike our study, Marlow et al. and Seung Cha et al., in their studies, neither the subjective nor objective clinical outcomes demonstrated the superiority of either plate system. This may be due to the involvement of all fracture types of distal radius not only comminuted intra-articular fractures (Table 4) [15, 16].

The correlation between the functional outcomes and the radiographic appearance of the wrist after a distal radial fracture remained debatable, as many studies reported that there is no correlation between both [18, 19]. This controversy may be due to the wide spectrum of injury patterns and different methodologies used by different investigators.

A recent study by Nishiwaki M. et al. compared the functional and radiographic outcomes of both types of plates and used a CT scan assessment at 6 months to evaluate the reduction quality and the plate prominence. Both simple and comminuted intra-articular fractures were included. They concluded that, despite having similar functional and radiographic outcomes, the VAP may be more prone to technical errors, leading to complications, whereas the FAP is more likely to require supplementary fixation (Table 4) [17].

In a prospective comparative study, Zenke Y. et al., compared the clinical and radiographic outcome of 118 patients with distal radius fractures fixed by volar locking plates, with and without associated ulnar styloid fractures. They found that there were no significant differences between both groups. In five (4.2%) cases with persistent ulnar-sided wrist pain, they thought it was related to relatively higher ulnar variance in these cases. These findings are consistent with ours as the results did not change when comparing both groups including and excluding cases with ulnar styloid fractures [20].

The management of distal radius fractures in osteoporotic patients is a hot topic in the literature. Although osteoporotic changes may start after the age of 50 years old, most of the studies in the literature use the age of 65 years old as a cut-off point to define fragility fractures in elderly patients [21–23]. None of our cases fall in this age group as the age of patients in our series ranged from 22 to 60 years. Twenty-six cases (27.1%) were in the age group (50–60 years), and most of patients in our series were younger than 50 years old (70 cases = 72.9%), yet the majority had fractured their distal radius following FOOSH (45 patients = 46.9%). High energy trauma including MVA, RTA, and FFH were the cause of injury in 33 cases (34.4%). This means that fractures in younger patients are not always the result of high energy trauma and extensive soft tissue damage is not frequently present in these cases.

Although old age is an important predictor of poor outcome after management of distal radius fractures, we still believe that inclusion of older cases (50–60 years) did not have an impact on the results of our series.

Several authors described flexor tendon complications following volar plating of distal radius fractures. This occurred as a late event in 4.3% of patients according to Soong et al. series. Fifty percent of the reported cases in the literature occurred within six to 26 months after the operation due to irritation (flexor tenosynovitis) with subsequent partial or complete rupture [24–26].

**Fig. 6:**
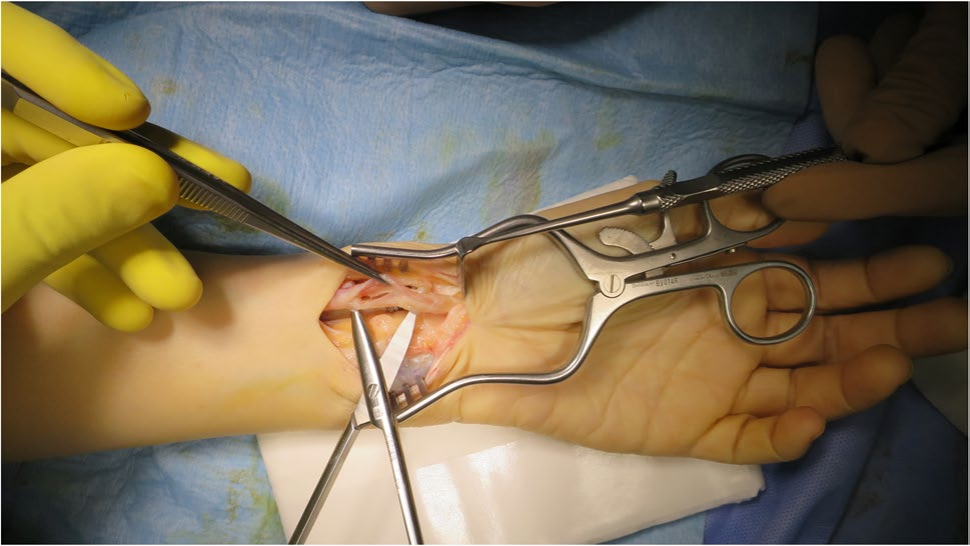
A case with frayed FPL tendon due to repeated friction with a plate which was placed distal to the watershed line

In our series, there were two patients with flexor tendon rupture. The first one had the flexor digitorum profundus (FDP) index ruptured at 11 months post-operatively, and this was related to a prominent proximal screw that was misplaced in an incomplete locking mode with about 2 mm prominence beyond the locking hole. The patient had a successful tendon transfer (extensor carpi radialis longus (ECRL) to FDP). The second patient developed flexor pollicis longus (FPL) rupture 26 months post-operatively (Fig. 6). The plate was prominent distal to the watershed line (Soong type 2). The patient had a successful tendon transfer (flexor digitorum superficialis of the ring finger (FDS-IV) to the distal stump of FPL).

These two cases did not add to our judgment about which volar locking system related to tendon rupture, as both of them (especially the first one) are related to a technical error that is totally avoidable. The attritional rupture of FPL (like the second case) may add to the fact that fixed angle plate is less forgiving when it comes to malposition.

There were some limitations for this study; in the VAP group, we used two different plate designs by different manufacturers, which potentially account for some disparity in results. The surgeries were performed by different surgeons. Although the patients in the two groups were comparable, they were not age and sex-matched.

The use of augmentation K-wires for radial styloid fixation in some of the FAP group cases might have influenced the data in many aspects. The presence of k-wires may have delayed the rehabilitation. Also, K-wires augmentation of the distal radius fixation raises an argument about the extra stability offered in addition to the plate with consequent unfair comparison to the unprotected VAP. However; the need for K-wires may reflect the inability of the FAP to catch the fragments as the screws could not be directed to purchase the radial styloid fragment, and this is not the case in VAP design.

Strength points in this study are that all patients had comminuted intra-articular fractures (AO 23 – C), both subjective and objective outcomes were used during follow-up giving a clear view about the progress, and the final data analysis was investigated by a single assessor.

## Conclusion

In this study, the overall functional results were in favor of the variable-angle plate, however; the rate of complications and reoperation were similar for both groups. Our recommendation is selective utilization of variable-angle plates in cases where screw angle require free decision to fix unstable bony fragments such as in distal radial fractures AO 23 –C2 and C3 and also with C1 distal radial fractures associated with styloid radial fracture or die punch fracture to fix these fragments in variable-angle mode.
